# Le carcinome verruqueux de la vulve: à propos de quatre cas

**DOI:** 10.11604/pamj.2014.17.303.2560

**Published:** 2014-04-21

**Authors:** Majdouline Boujoual, Hafid Hachi, Mouna Rimani, Basma El Khannoussi, Abdesslam Bougtab

**Affiliations:** 1 Gynécologie Obstétrique, Faculté de Médecine Oujda, Rabat; 2 Chirurgie Institut National d'Oncologie, Rabat; 3 Centre d'Anatomie Pathologique Hassan, Rabat; 4 Anatomie Pathologique Institut National d'Oncologie, Rabat

**Keywords:** Carcinome verruqueux, Anatomopathologie, chirurgie, verrucous carcinoma, pathology, surgery

## Abstract

Le cancer verruqueux de la vulve est une variante rare et bien différenciéedu carcinome épidermoïdedont les particularités diagnostiques, thérapeutiques et pronostiques méritent d’être distinguées. Il se caractérise par son importante croissance exophytique sans infiltration de la membrane basale. Son évolution est surtout locale rarement métastatique. Il est l'indication d'un traitement chirurgical exclusif avec exérèse large sans curage ganglionnaire de principe. La radiothérapie n'apporte aucun bénéfice en survie. Le pronostic est relativement bon mais grevé par les récidives locales. Nous rapportons quatre cas de carcinome verruqueux de la vulve colligés au Service de Chirurgieà l'Institut National d'Oncologie Rabat, nous discuterons à travers une revue de littérature, leurs modalités diagnostiques thérapeutiques et évolutives.

## Introduction

Les cancers de la vulve représentent moins de 1% de l'ensemble des cancers génitaux de la femme, ils touchent le plus souvent la femme ménopausée de plus de 60 ans [1]. Le cancer verruqueux est une entité rare constituant moins de 1% des cancers vulvaires, se caractérise par un aspectexophytique et une croissancelente rarementmétastasiantau niveau ganglionnaire [2]. En effet, c'est une forme bien différenciée de carcinome épidermoïde liée à l'infection virale par HPV 6, ayant l'allure de condylome géantsans envahissement de la membrane basale. Il a une évolution surtout locale, représentedonc l'indication d'un traitement chirurgical exclusif avec résection locale en zone saine sans contrôle ganglionnaire inguinal systématique [3, 4]. Nous rapportons quatre cas de carcinome verruqueux de la vulve, pris en charge en service de Chirurgie à l'Hôpital National d'Oncologie Rabat (INO), reflétant les particularités diagnostiques et thérapeutiques de cette pathologie.

## Méthodes

Notre étude est une analyse rétrospective portant sur 4 cas de carcinome verruqueux vulvaire colligés au service de Chirurgie INO entre 2004 et 2012. Notre analyse s'est penchée sur les caractéristiques cliniques, anatomopathologiques et thérapeutiques.

## Résultats

**Figure 1:**
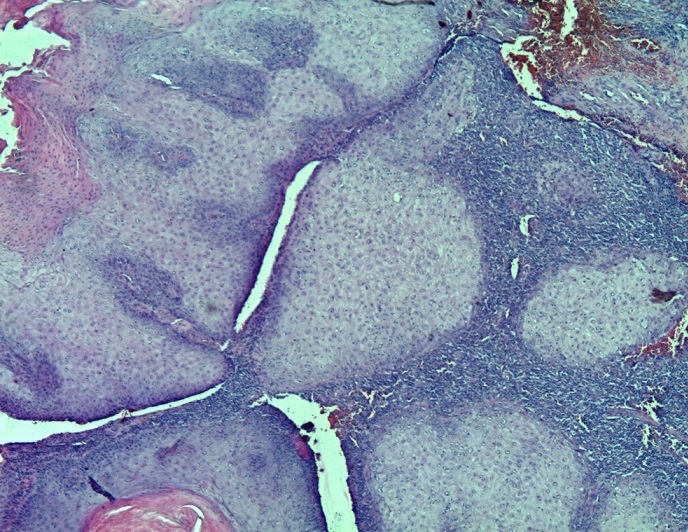
Coloration HEx50 montrant des projections globoïdes dès le derme

**Figure 2:**
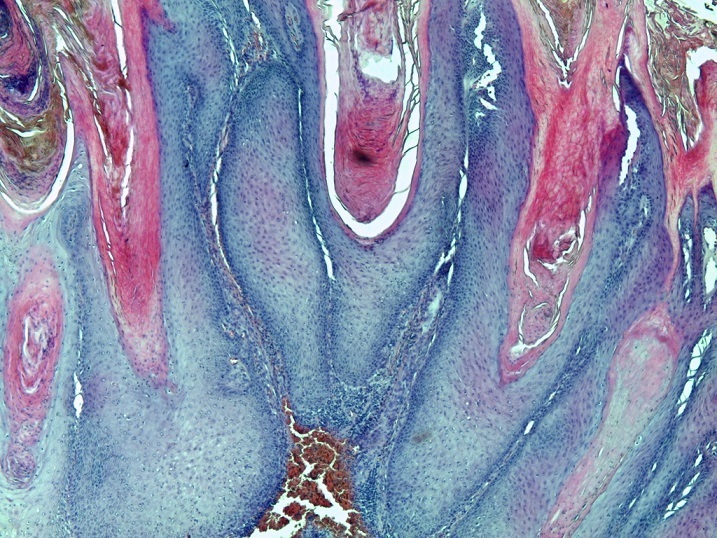
Coloration HEx50 montrant une proliferation carcinomateuse exophytique avec excroissances et invaginations

**Figure 3:**
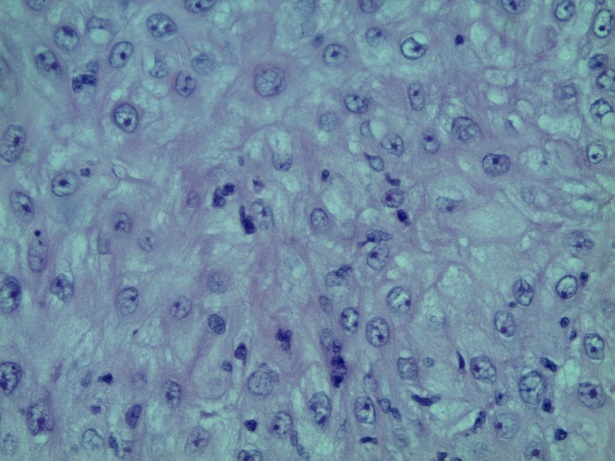
Coloration HEx200 montrant des cellules carcinomateuses avec atypies légère

**Figure 4:**
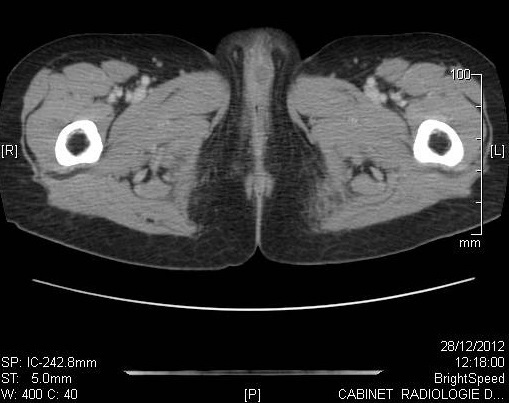
TDM abdomino-pelvienne montrant la présence au niveau de la vulve d'une formation tissulaire de 15mm de diamètre avec petit foyer central hypo dense en rapport avec une zone nécrotique et des adénopathies inguinales infra centimétriques

**Figure 5:**
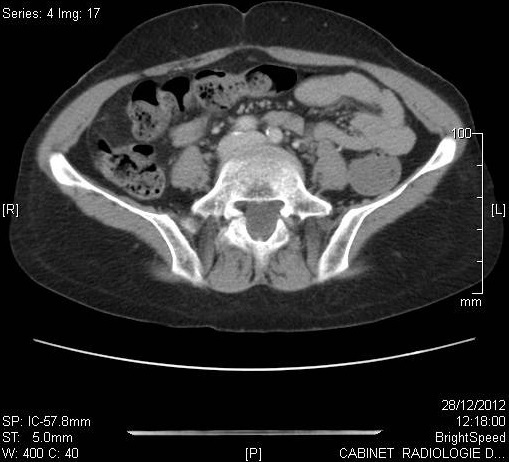
TDM abdomino pelvienne montrant la présence de ganglion inter aortico-cave de 9 mm et latéro aortiques gauches de 4 à 6 mm

**Figure 6:**
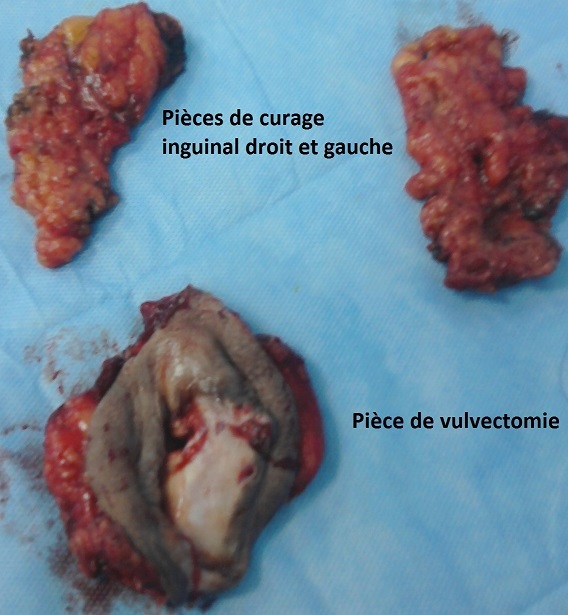
Pièce opératoire de vulvectomie totale avec curage inguinal bilatéral (cas N°4)

L′âge moyen au moment du diagnostic était de 61 ans avec des extrêmes de 54 et 68 ans. Toutes nos patientes étaient grandes multipares et ménopausées. Les symptômes initiaux les plus fréquents étaient un prurit et/ou une tuméfaction vulvaire. Le délai de consultation variait de 2 mois à deux ans avec une moyenne de 10 mois. Dans 2 cas, la lésion siégeait sur une grande lèvre, dans les autres cas sur la commissure ano-vulvaire. La taille moyenne des lésions était de 4,5 cm de diamètre (Tableau 1). L'examen anatomopathologique (Tableau 2) a montré un carcinome verruqueux bien différencié dans tous les cas (Figure 1, Figure 2, Figure 3), les ganglions étaient indemnes dans les cas N°1, 2 et 4. Toutefois, ils étaient atteints de carcinome épidermoïde bien différencié et mature dans le cas N°3. Le traitement consistait en une vulvectomie totale d'emblée avec curage inguinal bilatéral complété par une radiothérapie externe dans les cas N° 1 et 2, et une vulvectomie totale complétée par un curage inguinal et une radiothérapie dans le cas N°3, ceci est justifié par le stade avancé de la maladie et les berges d'exérèse atteintes ou limites. Dans le cas N° 4, le traitement initial était une exérèse large de la tumeur. Toutefois, le caractère envahi des berges d'une part,la persistance d'une tumeur résiduelle et la présence d'adénopathies inguinales, inter aortico-cave et latéro aortiques sur la TDM abdomino-pelvienne (Figure 4, Figure 5), nous ont conduits à compléter l'acte chirurgical par une vulvectomie totale et un curage inguinal bilatéral (Figure 6).

**Tableau 1:** Caractéristiques cliniques des tumeurs

Cas cliniques	Taille	Localisation	Aspect de la tumeur
Cas N° 1	3 cm	Commissure postérieure de la vulve + extension au 1/3 inférieur du vagin	Ulcéro bourgeonnant
Cas N°2	4 cm	Commissure ano vulvaire postérieure +infiltration anale	Ulcéro bourgeonnant
Cas N°3	7.5 cm	Grande lèvre	Ulcéro bourgeonnant
Cas N°4	3.5 cm	Grande lèvre	*Bourgeonnant verrucoïde*

Durant la période de 2004 à 2012, 4 cas de carcinomes verruqueux vulvaires ont été colligés au service de chirurgie INO, la taille moyenne des tumeurs était de 4.5 cm, l'aspect bourgeonnant était prédominant

**Tableau 2:** Particularités anatomo pathologiques et thérapeutiques

Cas cliniques	Traitement chirurgical	Anatomopathologie	Compléments thérapeutiques
Cas N° 1	VT+ CIB	CV bien différencié limite d'exérèse à 1 mm, N0	RTH sur la vulve (46 Gy)
Cas N°2	VT emportant quelques fibres du sphincter anal+ réfection sphinctérienne+ CIB	CV bien différenciéLimite d'exérèse profonde atteinte, N0	RTH vulvaire, inguinale et périnéale.
Cas N°3	VT	CV dans un contexte inflammatoire+ leucoplasie de voisinage, limite d'exérèse gauche atteinte	Reprise chirurgicale inguinale (10N+: CE différencié et mature)RTH vulvaire et inguinale
Cas N°4	Exérèse large de la tumeur	CV bien différencié+ limite d'exérèse profonde < 0.5 mm, stade pT1b	VT + CIB : absence de résidus tumoral post chirurgical, N0 : les ganglions siègent d'une histiocytose sinusale et parenchymateuse.

VT: Vulvectomie totale, CIB: curage inguinal bilatéral, CV: carcinome verruqueux, RTH : radiothérapie externe, CE : carcinome épidermoïde.

Par ailleurs, l’étude anatomopathologique a montré l'absence de résidus tumoral post chirurgical ni d'envahissement des ganglions qui étaient le siège d'une histiocytose sinusale et parenchymateuse. Les suites post opératoires ont été marquées par la survenue de lymphoedème des membres inférieurs dans le cas N°1, une importante radioépithélite dans le cas N°2, et l'apparition d'un condylome acuminéqui a été réséqué dans le cas N°3. Aucune récidive locale, locorégionale ou générale n'a été notée au cours de 24 mois.

## Discussion

Le carcinome verruqueux est une variété histologique rare, seuls 68 cas ont été rapportés dans la littérature, se caractérise par des particularités diagnostiques et pronostiques qu′il faudrait connaître pour adapter l′attitude thérapeutique. En effet, il peut survenir au niveau de la vulve, la sphère ORL, le pénis, le scrotum, ou le rectum [2, 5]. Il est localement invasif mais rarement métastasiant, atteint habituellement les femmes âgées en post ménopause, ce qui concorde avec notre étude. Toutefois, son incidence est en augmentation actuelle chez les femmes jeunes [2]. En effet, c'est une forme bien différenciée de carcinome épidermoïde lié à l'infection virale par l'HPV 6, retrouvé dans plus de 50% des cas [3, 5], parfois associé à une atteinte polyvirale, ce qui explique l'association fréquente du carcinome verruqueux de la vulve aux néoplasies intra épithélialesetau carcinome invasif de la vulve [5]. Son aspect est caractérisé par son importante croissance exophytique [6]: il s′agit d′une lésion bourgeonnante, en chou-fleur, parfois associée à une ulcération superficielle. Cet aspect peut faire porter à tort le diagnostic de condylome acuminé ou de carcinome épidermoïde bien différencié [7]. Ainsi, Une biopsie trop superficielle n′incluant pas le stroma sous-jacent peut sous-estimer la lésion donnant un diagnostic erroné de condylome acuminé ou la surestimer donnant à tort un diagnostic de carcinome épidermoïde d′un autre degré de différenciation, ces données de la littérature concordent avec le cas N°3. Il est par conséquent indispensable que la biopsie comporte toute l′épaisseur de la lésion: l′épithélium et une quantité suffisante de chorionafin d’éviter un diagnostic et un traitement inadéquat [2, 5]. L′examen microscopique montre une hyperplasie avec hyperacanthose, papillomatose et parfois hyperkératose de surface. Les atypies cellulaires et les mitoses sont rares. Des bourgeons épithéliaux sont ramifiés mais arrondis refoulent le chorion sous-jacent. Quant à la membrane basale, elle est toujours respectée [7].

Le traitement des carcinomes verruqueux de la vulve diffère de celui proposé pour les carcinomes épidermoïdes [5]. Toutefois, bien qu'il soit toujours un sujet de discussion [2], le carcinome verruqueux de la vulve est l'indication d'un traitement chirurgical exclusif même en cas de récidive. En effet, l'exérèselargeà marge centimétrique sans curage de principe est le traitement de référence en raison de sa malignité locale atténuée. Toutefois, l′extension des lésions peut néanmoins rendre le geste délabrant, c'est le cas des patientes N°1 et 2. Ainsi, le curage ne peut être justifié que si l'examen définitif révélait la présence d'une zone invasive vraie [3, 5], puisqu'il expose aurisque de complications telles que lymphocèle (40%), infection de cicatrice (39%), lymphoedème (28%) et désunion de suture (17%) [8]. Par ailleurs, la radiothérapie n′apporte aucun bénéfice en survie. Elle peut même induire une transformation anaplasique post radique avec apparition de lésions à potentiel métastatique élevé. Le pronostic est relativement bon en cas d'excision large mais grevé par les récidives locales et le risque d'apparition de localisations secondaires en cas de persistance des facteurs étiologiques (Lichen scléro atrophique ou HPV) [2, 4, 5]. Ce ci justifie une surveillance clinique tous les 4 mois au cours de la première année, tous les 6 mois pendant 2 ans puis tous les ans avec un examen clinique de la vulve, périnée et aires ganglionnaires,éventuellement complété par une échographie, cytoponction et frottis de dépistage [4].

## Conclusion

Les carcinomes verruqueux sont donc une forme histologique rare bien différenciée du carcinome épidermoïde qui se distingue par ses modalités diagnostiques, thérapeutiques et évolutives particulières. Son traitement est exclusivement chirurgical se base sur une excision large sans curage ganglionnaire. Le pronostic de ces lésions est bon mais grevé par les récidives locales.
